# Macroscopic findings of the skull and brain in fire fatalities - an update

**DOI:** 10.1007/s12024-025-01058-9

**Published:** 2025-08-06

**Authors:** Michael Bohnert, Helmut Heinsen, Pawan Mittal, Simone Bohnert

**Affiliations:** 1 Institute of Forensic Medicine, Julius-Maximilians-Universität, Versbacher Str. 3, 97078 Würzburg, Germany; 2 Department of Forensic Medicine, N.C. Medical College and Hospital, Panipat, India

**Keywords:** Brain, Skull, Burns, Fire death, Macromorphology

## Abstract

On the skull, the best known and most frequently observed autopsy findings are the heat fractures of the skull and the burn hematoma. The macromorphological changes of the brain have been described comparatively less frequently in the primary and secondary literature. We have evaluated the fire deaths of our institute over a period of 10 years with regard to the macromorphological changes of the skull and brain and described four stages (0-III) after postmortem fireexposure. In stage 0, the scalp showed up to third-degree burns, but the skull and brain were not affected. In stage I, in which the scalp showed fourth degree burns but the skull was still completely intact and unbroken, a certain hardening of the brain and a paling of the lateral parts of the temporal lobes, in particular, could already be regularly observed. In stage II, the head skin was burned away, and the skull was charred but still closed. There was a noticeable shrinkage of the dura mater, which was often torn. The brain itself appeared shrunken, pale and showed a flattening of the convolutions and a spreading of the furrows. In stage III, the skull was opened, charred to calcified and broken in clods. Tears in the dura mater led to herniation of brain tissue to the outside. The aim of this study was to classify the macromorphological changes of the skull and brain after fire exposure, both chronologically and according to their severity. The proposed staged classification (0-III) is intended to summarize the findings and to update and complement the descriptions in the older literature. It can be seen as an extension and further development of the Crow-Glassman scale, which classifies the extent of fire destruction of human bodies.

## Introduction

The external and internal findings in cremated corpses are characterized by a large number of predominant post-mortem thermal artifacts like pugilistic attitude, skin splits, protrusion of the tongue, charring and destruction of the corpse [1–6]. The best known autopsy findings at the head are the heat fractures of the skull [7–10] and the so-called “burn hematoma” [7, 11–13] which is a brick-red bloody material that extrudes from the diploë or results from vascular tears, and accumulates between the tabula interna of the skull bone and the underlying dura mater.

The macromorphological changes in the post-mortem fire exposure as well as the clarification of the question of whether basal skull fractures are caused by a heat-induced increase in pressure in the skull bone, could be described and clarified by observations in the crematoria [14]. In contrast, macroscopic changes in the brain itself after post-mortem exposure to fire are described comparatively rarely in the scientific literature. These are mostly individual case reports with unusual findings [15–18]. While the old specialist literature contains differentiated descriptions of the external and internal organs, especially in the casuistic descriptions of fire deaths (e.g. [19–21]), the description of the brain is usually rather superficial. A representative example is Böhmer’s publication from 1932, in which the following is quoted from an autopsy protocol: “Brain transformed into a greasy mass whose proper autopsy is no longer possible” [19]. Even in the textbooks, there are only brief descriptions of the brain, such as in Spitz: “The brain may be shrunken, firm and yellow to light brown, due to boiling and desiccation” [7]. The description in Knight and Saukko’s textbook is somewhat more detailed, who report the following in connection with epidural burn hematoma: “Where the heat is intense, the spurious haematoma may be outside a grossly shrunken dura, which compresses the brain into a cooked mass. The dura may split under tension and allow brain tissue to ooze out into the large space within the cranium, where it may form a mass of frothy paste” [13].

The most comprehensive study on heat-induced changes in the brain was published by Dotzauer and Jacob in 1952 [22]. It described the consistency and coloration of brain tissue according to the intensity, duration and extent of the burning process. The authors reported that the brain exhibited the characteristics of a so-called doll’s brain with the most severe charring of the scalp and the skull, which could be attributed to water evaporation, boiling and stewing processes as well as to the tissue dehydration and could ultimately lead to attrition and even total ashing of the tissue. In this context, a spreading of the surface relief with flattening of the convolutions of the brain was described in the context of its heat-induced shrinkage, as is also found in the brain oedema. An initial swelling of the brain as a direct reaction to the heat with subsequent shrinkage or a coordinated swelling of the substance with a simultaneous reduction in the volume of the brain were discussed as pathomechanisms. Remarkable in this work are the detailed histological descriptions of the brain. The authors referred to the preservation of the histological structure of the internal organs, particularly the brain and heart. The observation of heat-induced tissue fixation with good visualization of the cortex and medullary layer was recently confirmed in the neuropathological examination of two fire fatalities [23].

While histopathological and cytopathological findings on the brain have been updated in the literature [23], there are no contemporary descriptions of the macromorphological changes of the skull and brain in burn-related deaths. Furthermore, there is no categorization of the macroscopic changes of the head. The known stagings of burnt corpses only partially address the destruction of the head [24–26]. For this reason, the present study retrospectively analyses the fire fatalities examined in our institute over a duration of ten years, covering the period from 2014 to 2023 inclusive, in order to give a description of the macromorphological findings, depending on the degree of burns to the skull and brain. We propose a staging (0-III) to classify the heat-induced changes to the skull and brain, according to their severity and to update and complement the descriptions in the older literature.

## Materials and methods

In the 10-year period from 2015 to 2024 inclusive, 46 fire fatalities were autopsied at our institute (1% of all autopsies), 11 of which were excluded from further investigation as they were pure smoke inhalation without signs of direct heat exposure to the body. The remaining 35 cases were 30 male and 5 female deceased, aged between 20 and 93 years (MW: 61 years, median: 58 years). In three cases, there was only postmortem exposure to fire; in all other cases, signs of vitality could be detected during autopsy. Two cases were homicides with attempted burning of the body, one case was a suicide by a shot to the heart, followed by a house fire. The autopsy protocols and photographs were analyzed retrospectively and independently by 2 investigators and the results were compared with each other. In case of discrepancies, the findings were submitted to a third examiner for evaluation. The following parameters were analyzed: Classification according to Crow-Glasmann-Scale, degree of burns/burns of the scalp, condition of the skull, presence of a heat hematoma, condition of the dura mater, weight of the brain, condition and consistency of the brain. The evaluation revealed recurring patterns that suggested a classification into four stages (0-III).

## Results

The retrospective analysis of the fire fatalities revealed a total of 4 stages that could be clearly distinguished morphologically. The findings are summarized in Table 1.

**Table 1:** Case characteristics and staging

Nr.	Sex	Age (years)	CGS grade	Stage	Brain weight (g)	Burn hematoma	Circumstances
1	male	76	1	0	1265	no	House fire
2	male	79	1	0	1470	no	House fire
3	male	85	1	0	1450	no	House fire
4	male	76	1	0	1430	no	House fire
5	male	64	1	0	1490	no	House fire
6	female	81	1	0	1320	no	House fire
7	male	49	1	0	1290	no	House fire
8	female	41	2	0	1325	no	Homicide by sharp and blunt force, post-mortem burning
9	male	54	2	0	1465	no	Fire following car accident
10	male	63	2	0	1425	no	House fire
11	male	41	2	I	1425	yes	Suicide by self-immolation in a car
12	male	57	2	I	1650	no	Self-immolation in the open air
13	male	76	2	I	1250	no	House fire
14	male	93	2	I	1260	no	House fire
15	male	87	2	I	1260	no	House fire
16	male	44	2	I	1530	no	House fire
17	male	77	2	I	1380	no	House fire
18	female	52	2	I	1125	no	Car fire
19	male	58	2	I	1310	no	House fire
20	male	66	2	I	1600	no	House fire
21	male	55	3	I	1170	no	House fire
22	male	61	3	I	1440	no	Suicide by self-immolation on a pyre
23	male	90	3	II	1210	no	House fire
24	male	82	3	II	1120	no	Airplane crash
25	male	64	3	II	1200	yes	House fire
26	male	43	3	II	1250	yes	House fire
27	male	56	3	II	1400	yes	House fire Post-mortem burning after shot to the heart
28	male	88	3	III	-	yes	Suicide by self-immolation on a pyre
29	female	52	4	III	-	yes	Suicide by self-immolation in a car
30	male	52	4	III	-	no	Airplane crash
31	male	51	4	III	-	not detectable	Suicide by self-immolation in a car
32	male	54	4	III	1055	no	Car fire
33	male	28	4	III	-	not detectable	Fire following car accident
34	female	55	4	III	1060	yes	Homicide by strangulation House fire
35	male	20	4	III	937	yes	Car fire

In **stage 0** (10 cases), the scalp showed up to third-degree burns, but the skull, the dura mater and the brain showed no changes that could be considered heat related. There were no differences to non-fire deaths. There were no burn hematomas. The mean brain weight was 1393 g.

**Fig. 1:**
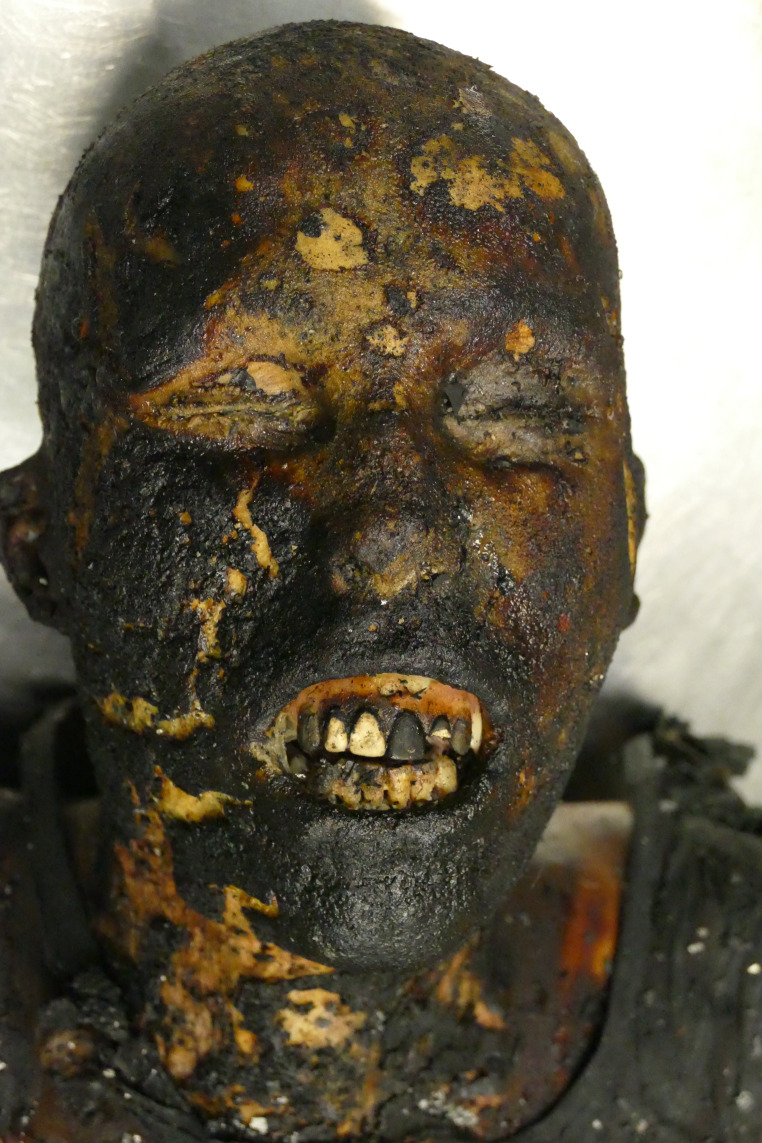
Stage I: Third to fourth degree burns of facial soft tissues and scalp with preserved skull (55-years-old male, house fire)

**Fig. 2:**
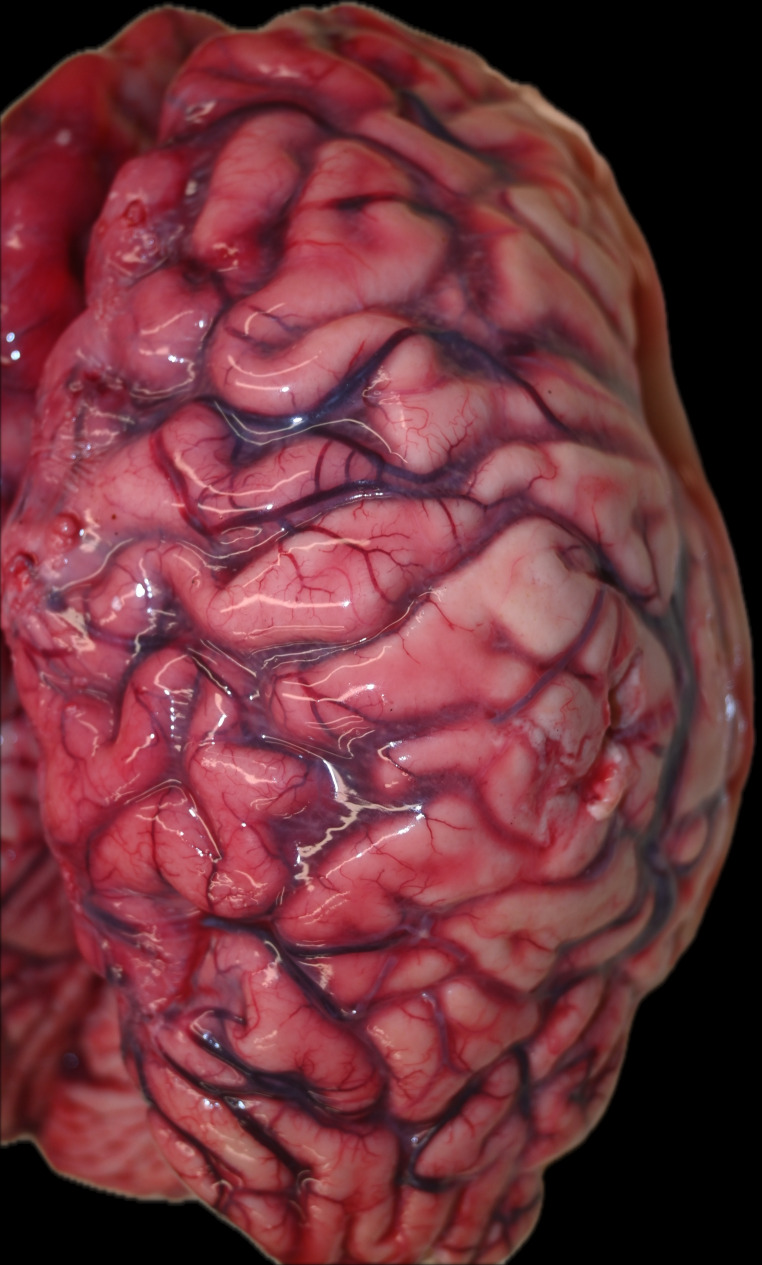
Stage I: Right hemisphere with localized pallor of the gyri at the lateral parts of the brain. Surrounding cortex shows signs of congestion

In **stage I** (12 cases), there were up to fourth degree burns of the scalp, however, the skull was still completely covered (Fig. 1). The skull exhibited no additional morphologically discernible alterations. In one case, a thin, liquid accumulation of blood was observed in the epidural space, without evidence of a calvarial fracture. In all other cases, epidural or subdural accumulation of blood could not be detected. Circumscribed regions of cortical tissue induration could be verified by palpation. They coincided with pale gyri in the ventral central and lateral parts of the frontal/parietal and the temporal lobe, respectively (Fig. 2). It is noteworthy that the brain stem and cerebellum appeared unaffected. The average brain weight was 1360 g.

**Fig. 3:**
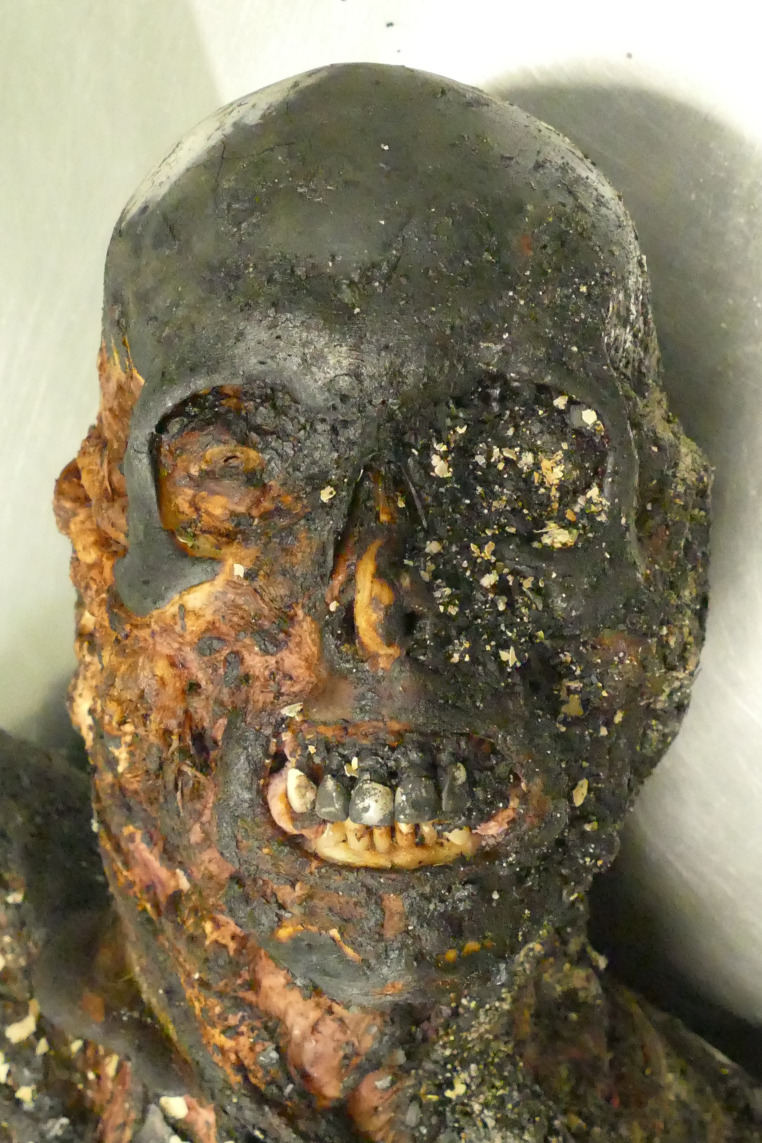
Stage II: Burnt-away scalp reveals a charred skull with calcined right parietal bone. Early stellate thermal fractures of the right frontal bone (64-years-old male, house fire)

**Fig. 4:**
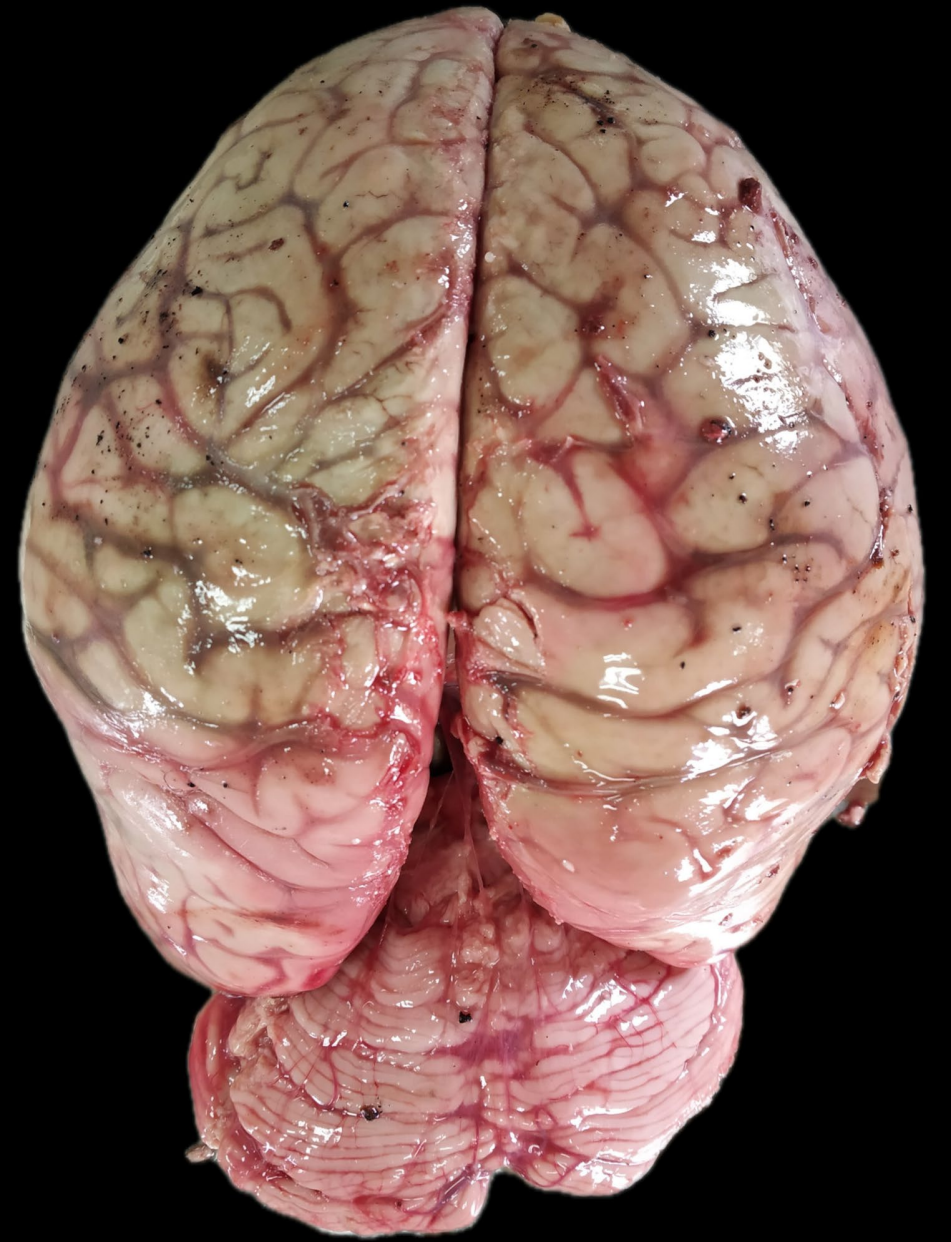
Stage II: Cortical pallor with flattening of the convolutions like in a cerebral edema, but with solidified consistency of the brain. Pink discoloration of occipital cortex and cerebellum (23-year-old male, closed hostel room fire)

In **stage II** (5 cases), the scalp was at least partially burned away, and the exposed skull was charred (Fig. 3). The dura mater had shrunk and detached in places from the skull or the base of the skull. In 4 of the 6 cases, it was torn. A burn hematoma was present in half of the cases. The brain itself appeared shrunken, pale and showed a flattening of the convolutions as well as a widening of the furrows as in cerebral edema (Fig. 4). However, the tissue was not soft, but solidified and had a dry, crumbly, somewhat greasy consistency. The medullary layers were rather pale; the cortex was grayish pink in color. The average brain weight was 1236 g.

**Fig. 5:**
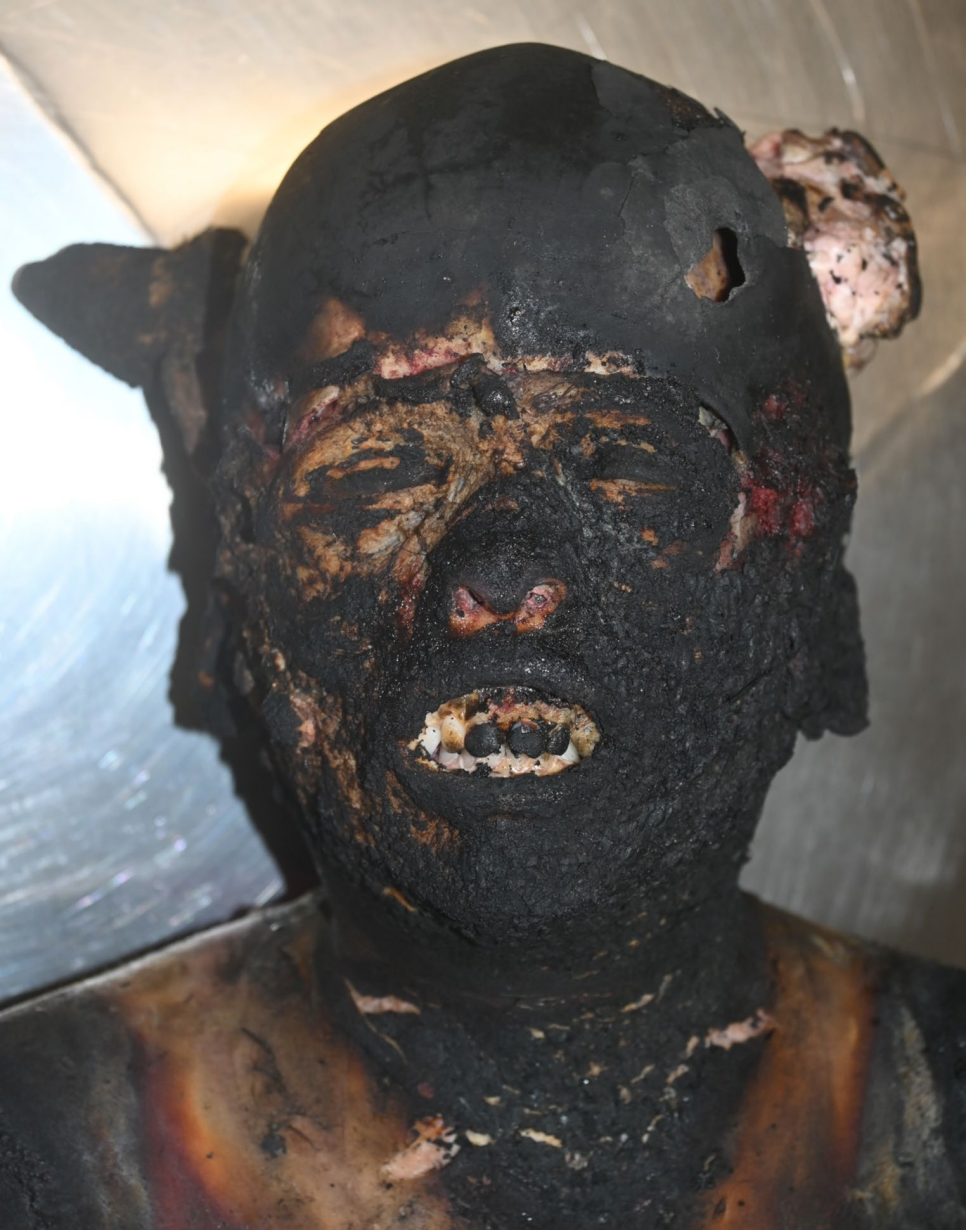
Stage III: Scalp and soft tissue loss exposes charred skull. Fragmented left parietal bone reveals herniation of brain tissue (20-years-old male, car fire)

**Fig. 6:**
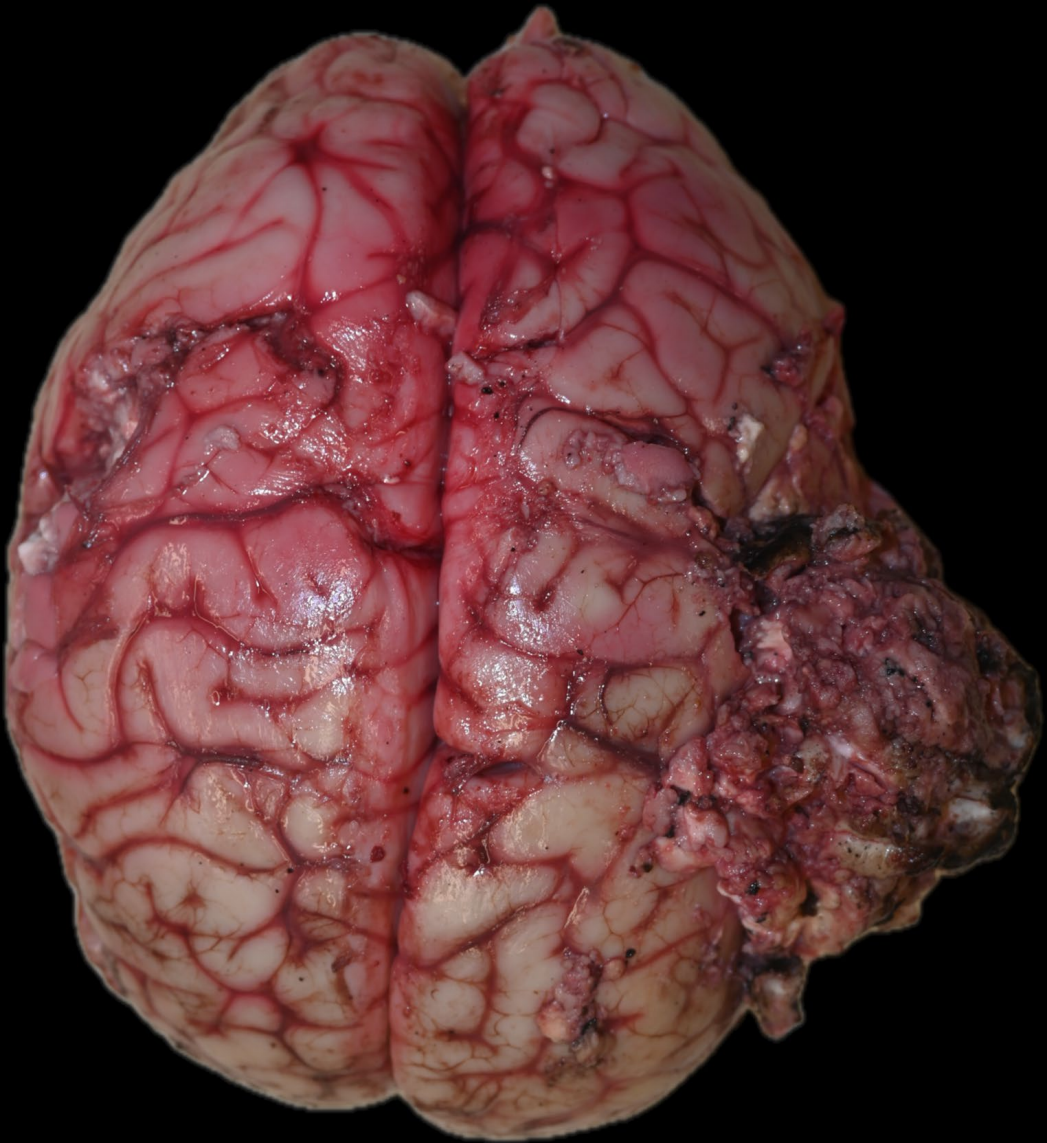
Stage III: Cauliflower-like extrusion of the left parietal-temporal brain through torn dura and fragmented skull. The great majority of the cortex appears pale gray to gray-pink

In **stage III** (8 cases), the scalp was burned away, the skull was charred to calcined and broken in clods, so that the cranial cavity was open (Fig. 5). A burn hematoma was present in 6 of the 8 cases. The dura mater was partially detached from the inner table of the skull and in places from the skull base and torn, which led to herniation of brain tissue to the outside (Figs. 5 and 6). In half of the cases, large parts of the brain had leaked out and could no longer be found. The brain was of crumbly to greasy consistency, the cerebral cortex was dirty grayish to grayish pink in color. The basal parts of the brain, especially the cerebellum and brain stem, were comparatively better preserved, with a softer consistency than the cerebrum. The cerebellum showed a grayish-pink discoloration of the cortex. The average brain weight was 1017 g, whereby in 5 cases the brain was only partially present or completely missing. These cases were not included in the calculation.

In three cases there was evidence of mechanical traumatization of the head. One was a homicide with stabs to the chest and blows to the head. This case was classified as CGS 2 and stage 0 in our classification. There were no fractures of the skull. One case was an airplane crash (CGS 4, stage III), where the skull was destroyed. Another case was a traffic accident (CGS 4, stage III) with similar findings.

## Discussion

In the proposed staging, this study summarizes the macroscopically visible changes to the skull and brain caused by heat, although these do not provide any information about vitality at the time of exposure to the fire and are independent of it. In any case, it can be assumed that they only develop after death has occurred. Rather, they indicate the great variability of possible findings on the skull and brain, as described, for example, in Glassman and Crow’s classification (CGS [26]) of the stages of burn injuries. Our proposed staging is compatible with the Crow-Glassman scale, even if there were small deviations in individual cases. Our stage 0 predominantly corresponded to CGS 1, in individual cases also to CGS 2. Our stage I predominantly corresponded to CGS 2, in individual cases also to CGS 3. This can be explained by the fact that the Crow-Glassman scale on the head only distinguishes between ‘skull intact’ (CGS 3) and ‘skull fragmented’ (CGS 4). The variety of morphological changes may also reflect the variable external conditions in individual cases, which in many cases cannot be clearly reconstructed. However, it probably makes a difference whether the corpse was in the center of a full fire, whether there was a circumscribed flame contact or whether the head was exposed to heat radiation or convection. The level of the acting temperature, the type of heat exposure and its duration as parameters suggest different constellations of findings.

The development of the findings in the case of a complete fire could be observed in cremations [14]. It could be seen that after 15 to 25 min, the scalp of all the deceased had burnt away and the cranial roof exhibited fractures or a bursting of the coronal suture, whereby these fracture lines began to gape in the following minutes. The tabula externa was calcified in places. After a further 10 min, boiling fluid could be observed oozing from the fractures. The top of the skull crumbled, and the shrunken brain became visible. It appeared as a “cooked mass, part of which was dripping out of the burst cerebral cranium as a viscous mass”. If these observations are transferred to the proposed stage classification, then stage II is reached after a full burn time of about 20 min and stage III after a full burn time of about 30 min. We are aware that this estimate can only be approximate and does not consider the highly variable conditions in individual cases. Nevertheless, the observations made during cremations can serve as a rough time reference for full fires [14, 27, 28].

The formation of the findings on the skull, when exposed to a circumscribed flame, was already described by Westenhoeffer in an individual experimental observation [29]. He found that when the scalp was burned away and the outer bone plate was charred, a foamy boiling liquid oozed out of the smallest openings. This ended when the diploe had completely dried out. After opening the skull, he found the dura mater “strongly expanded, smooth and intimately attached to the inner bony plate”. On direct exposure to flame, the dura mater tore, and a pulpy, grayish-reddish brain mass emerged from the tear under pressure, which corresponds to our observations in stage III and the herniation of tissue to the outside.

In his book “Tod im Luftangriff” [Death in Air Raid], Gräff described his observations of deceased persons who had died in the air raids on Hamburg in the years 1943–1945, many of them in the firestorms of the 1943 bombardments [30]. The heat-induced changes to the head are kept comparatively superficial in the case reports, but in the summary of the findings he described: “Lipoid-rich organs such as the brain or liver are homogeneously imbibed by fat, acquire a clay-like consistency in the transition and cut like cold butter”. Thus, in the classification presented, from stage I onwards, an incipient solidification of the tissue as well as a pallor, particularly of the lateral parts of the temporal lobes, could be observed, which could indicate a possible easier displacement of the blood in the lateral parts of the brain due to the thinner bony part of the temporal bone. In stage II, the solidification of the tissue was so pronounced that the brain even stood up when cut - reminiscent of cold butter - and did not have the usual soft and runny consistency.

In addition, Gräff explained that the dura mater was often missing due to the prolonged exposure of the body to heat, with the brain shrinking and becoming slightly charred. The loss of brain mass was up to 80% in some cases. This is consistent with individual case descriptions such as that of Westenhoeffer, who also described a generalized shrinkage of the brain (“It is reduced to about the fifth part of its volume, corresponding approximately to a large dog’s brain”) [29]. In the proposed stage classification, a generalized shrinkage of the brain was observed. In stage 0, an average brain weight of 1393 g was measured, while in stage III the average weight was 1017 g. Thus - as already mentioned - water evaporation, cooking and stewing processes as well as tissue dehydration led to the formation of a pupal brain, which was also described in the study by Dotzauer [1, 22, 30].

From stage II onwards, a burn hematoma could be detected in most cases in the proposed classification, which is to be expected above all when there was a direct and possibly circumscribed effect of the fire on the bony skull [1, 11, 31]. Gräff had not been able to find these extradural hematomas known to him from the literature in the burned corpses [30]. This discrepancy between the sometimes pronounced heat shrinkage of the tissues and the absence of burn hematomas may be because the deceased examined by Gräff had been exposed to great heat for a very long time, but not *directly* to a full fire.

The aim of this study was to classify the macromorphological changes in the skull and brain after fire exposure chronologically and according to their severity. The proposed staging (0-III) is intended to summarize the findings and to update and complement the descriptions in the older literature to provide suggestions for handling under routine and practical conditions. The staging is compatible with the Crow-Glassman scale and can refine it, particularly regarding the heat-induced changes of the skull. It may also be a tool for predicting the likelihood of intracranial findings like tearing of the dura mater, burn hematoma, and morphological changes of the brain which highlights the novelty of the proposed staging. The summary of the macroscopic findings of this study and the recently published microscopic changes in the brain are intended to serve as an “update” in the scientific literature.

### Limitations

The proposed stage classification (0-III) summarizes the macroscopic changes in the skull and brain after post-mortem fire exposure. The findings are based on a very heterogeneous sample with a wide age range and an unbalanced gender proportion, but this represents our autopsy material in the period from 2015 to 2024. Further studies are therefore necessary to verify the staging and to refine apparently unnoticed findings like small defects, thermal splits of the skull with larger case numbers and homogenization of the sample.

### Key points

Analysis of the macromorphological changes in the skull and brain after fire exposure revealed a total of 4 stages:

1. Stage 0: The scalp showed up to third-degree burns. The skull, the dura mater and the brain showed no changes.
2. Stage I: The scalp showed fourth degree burns but the skull was still completely covered.
3. Stage II: The scalp was burned away, the skull was charred but is still closed, the dura mater showed a noticeable shrinkage, and the brain appeared shrunken, pale and showed a flattening of the convolutions and a spreading of the furrows.
4. Stage III: The skull was opened and was charred to calcified and broken in clods. Tears in the dura mater led to herniation of brain tissue. The brain was of crumbly to creamy consistency.
